# Obstacles and Trials in Treating Primary Brain Tumors

**DOI:** 10.3390/ijms27167390

**Published:** 2026-08-18

**Authors:** Stefana Oana Popescu, Delia Codruta Popa, Oana Alexandru, Anica Dricu

**Affiliations:** 1 Department of Biochemistry, Faculty of Medicine, University of Medicine and Pharmacy of Craiova, Petru Rares Street No. 2–4, 200349 Craiova, Romania; oana.popescu@umfcv.ro; 2 Department of Hematology, Fundeni Clinical Institute, Soseaua Fundeni No. 258, 022328 Bucharest, Romania; delia.popa@umfcd.ro; 3 Department of Pediatrics (Laboratory Medicine), Faculty of Medicine, “Carol Davila” University of Medicine and Pharmacy, Bd Eroii Sanitari 8, 050474 Bucharest, Romania; 4 Department of Neurology, Faculty of Medicine, University of Medicine and Pharmacy of Craiova, Petru Rares Street No. 2–4, 200349 Craiova, Romania; 5 Department of Biochemistry, Faculty of Medicine, “Carol Davila” University of Medicine and Pharmacy, Bd Eroii Sanitari 8, 050474 Bucharest, Romania; anica.dricu@umfcd.ro

**Keywords:** brain tumor, neurological complications, therapy, strategies to manage adverse effects

## Abstract

Brain tumor (BT) patients can experience neurological complications as a result of the disease itself or after exposure to anticancer treatment. It is already known that BT patients have a poor survival and quality of life due to tumor progression, but also to various complications. The neurological complications may represent obstacles in patient management and require multidisciplinary collaboration. Although new methods of surgery, radiation therapy and pharmacotherapy have been designed in the last few years in order to prevent, mitigate, or manage the adverse effects of classical therapies, the overall survival of patients with BTs, especially glioblastoma (GB), remain poor. In this review we provide an overview of the most commo022328n neurological complications of BTs, as well as tumor therapies. We present what is already known on the topic but also the newest clinical data, and the results of clinical trials from the last few years. We also make a summary of the strategies capable of managing or even preventing these adverse effects.

## 1. Introduction

Primary BTs are capable of affecting the central nervous system (CNS) and generating a wide range of complications, including neurological ones. Although there is a connection between the location of the BT and its symptoms, there are no specific clinical signs that can be attributed to these entities. Usually, the symptoms have a slowly progressive evolution and are subacute. Most patients complain of headaches, nausea, vomiting, mental changes, gait disturbances, radicular neck and back pain, hemiparesis, aphasia, or cranial nerve palsies. An accurate neurological examination is required in each case. A quantitative assessment of neurological functions using Neurologic Assessment in Neuro-Oncology (NANO) criteria is also recommended. This scale assesses nine domains—gait, strength, ataxia, sensibility, visual field, facial paralysis, language, level of consciousness, and behavior—and is also useful in the patient follow-up process [1].

BT complications require multidisciplinary collaboration. Among the neurological complications observed in these patients are brain edema, seizures, neurocognitive impairment, cerebral venous thrombosis, stroke (ischemic or hemorrhagic), metastatic spinal cord compression, thrombotic microangiopathy, posterior reversible leukoencephalopathy syndrome, radiation-induced vasculopathy including stroke-like migraine attacks after RT (SMART) syndrome, Moya-Moya-like vasculopathy, and microbleeds. All these entities are described and have recommendations for their clinical management in the joint guidelines of European Association of Neuro-Oncology (EANO)-European Society for Medical Oncology (ESMO) [2].

BT treatment includes increasing modalities. Besides current standard of care like surgery, radiotherapy, and approved pharmacotherapy, nowadays new therapies are integrated into these patients’ treatment plans. However, the management of treatment complications is still a challenge for clinicians. There are complications of conventional treatments and of novel therapies, but also for long-term effects of anti-tumor interventions. In 2023, the European Association of Neuro-Oncology (EANO) developed recommendations to prevent, diagnose, and manage adverse effects and complications in adult primary brain CNS tumors [3].

In clinical practice, clinicians are confronted with the neurological complications generated by BT therapies. These complications impact on neurological function and on the quality of life of BT survivors, and presents a challenge nowadays. Because they are not properly studied, there is a lack of consensus regarding their prevention and management.

For instance, in GB patients an important issue is finding the equilibrium between maximum safe resection and the preservation of neurological functions. Modern neurosurgical techniques like image-guided surgery, image-guided navigation, fluorescence-guided surgery, awake craniotomy, and confocal laser endomicroscopy help neurosurgeons to better achieve this difficult goal [4]. There are also methods to prevent, mitigate, or even treat post-brain surgery complications like venous thrombosis, cerebrovascular events, seizures, and hydrocephalus.

Radiation therapy is another important method of treatment for BT patients. However, besides its essential role in controlling tumor growth, it may also induce acute and long-term neurological complications in these patients. Nowadays, there are new, modern irradiation methods like proton beam therapy, stereotactic radio surgery, intensity-modulated radiation therapy, and intraoperative radiotherapy which try to prevent or mitigate these complications, trying to preserve as much as possible the healthy brain structures and prevent the adverse effects of classical radiotherapy [5].

Chemotherapy is also a key treatment for patients with BTs. There are several neurological complications like myopathy, neuropathies, or cognitive impairment, which nowadays can be mitigated, treated, or even prevented [3].

Here, we present the obstacles generated by neurological complications of tumors and tumor therapies in patients with primary BTs. We also provide updated information about the neurological complications of BT therapies, as well as the novelty in the literature regarding the prevention and management of these complications.

## 2. Neurological Complications of Primary Brain Tumors

### 2.1. Brain Edema

Brain edema is a condition commonly found in malignant and benign BTs. This edema is vasogenic and extracellular, and is consequence of blood-brain barrier (BBB) disruption. For instance, from 40% to 92% of meningiomas present with peritumoral brain edema [6]. Gliomas are also surrounded by edema which promotes tumor invasion, indicates tumor spread, and predicts tumor grade, recurrence, and prognosis [7,8,9]. The mechanism of formation includes three possibilities: increased BBB permeability, tumor angiogenesis, and increased expression of aquaporin 4 [10]. As the tumor is growing, it secretes angiogenic factors such as vasculo-endothelial growth factor (VEGF), which binds through VEGF receptors to capillary endothelial cells, downregulating the tight junction expression and structure and increasing vascular permeability. VEGF is involved in vascular permeability and tumor angiogenesis [11]. Also involved in BBB permeability are matrix-metal proteinases (MMPs), cyclooxygenase-2 (COX-2), leukotrienes, nitric oxide, and mast cell activation, all leading to edema formation [12]. In gliomas and meningiomas, aquaporin 4 is overexpressed and also associated with peritumoral edema formation [13,14]. Growth factor receptors (GFRs) are overactive or mutated in more than 80% of aggressive high-grade gliomas (HGGs), inducing abnormal cell proliferation, blood vessel formation, and survival [15,16].

GFR inhibitors are more precise than standard treatments of gliomas, but frequently cause “on-target” side effects, inducing cytotoxicity responses in healthy cells [17].

The diagnostic tools are MRI or CT scans, and steroids remain the drugs of choice for these patients. However, because of their side effects (pneumonia, diabetes, arterial hypertension, osteoporosis, myopathy, psychiatric symptoms) when used long-term, they should be used only as long as there is clinical benefit [2].

Therefore, nowadays several other agents are studied in preclinical and clinical trials for peritumoral edema treatment.

The use of bevacizumab, a Food and Drug Administration (FDA)-approved monoclonal antibody that targets VEGF for glioma or meningioma treatment to reduce peritumoral edema, with the reduction of steroid doses, is supported by several clinical studies. Unfortunately, the overall survival did not improve in glioma patients. Also, the studies had limitations because they were single-center and retrospective studies [18,19].

**Figure 1:**
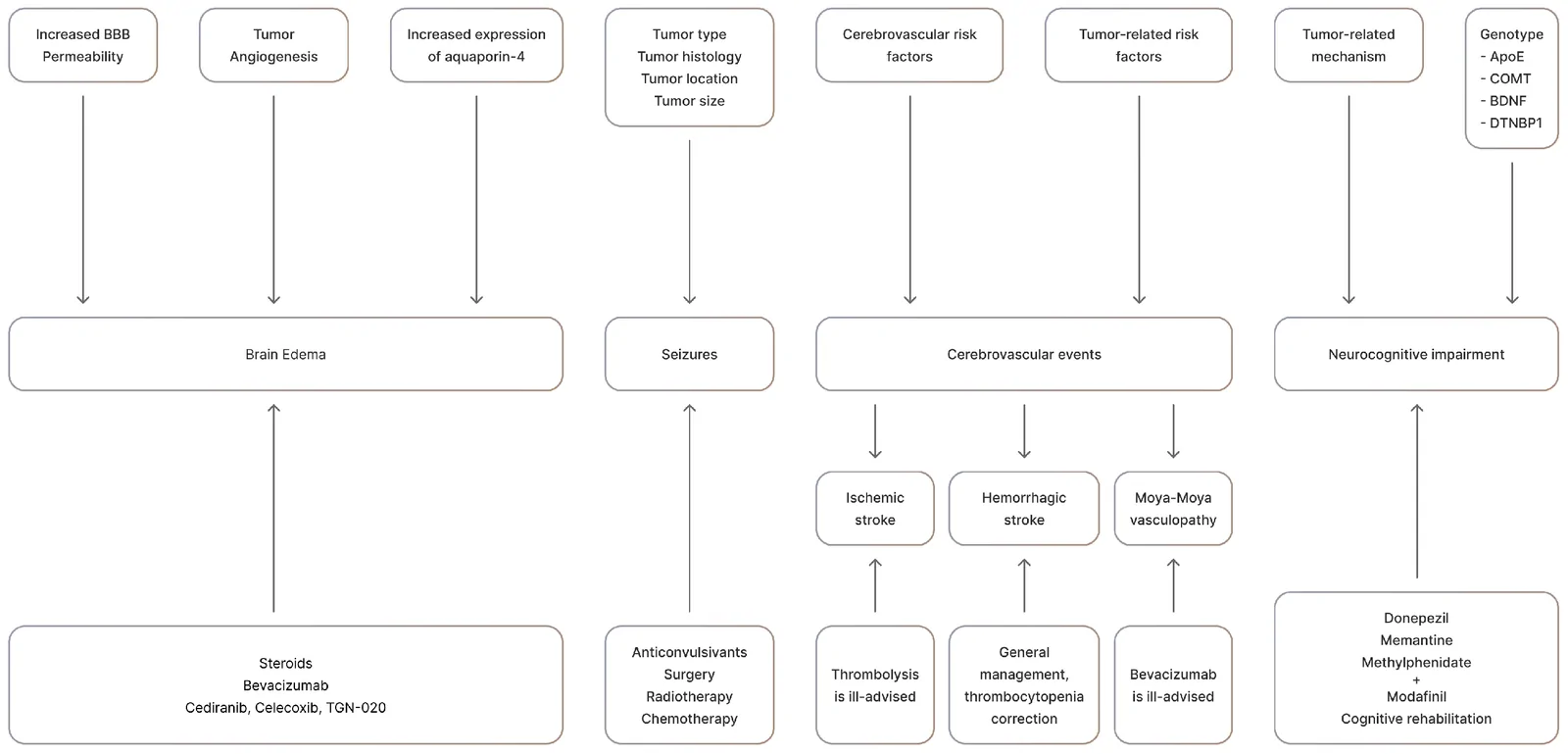
Neurological complications of BTs and their treatment.

Cediranib, another VEGF small-molecule inhibitor, showed promising results in reducing peritumoral edema in mice with BTs [20]. Celecoxib, a Cox-2 inhibitor, reduced peritumoral edema in animals with GB [21], but in clinical studies, further data is needed and the adverse effects were significant [22]. Aquaporin 4 inhibitors like TGN-020 or AER-271 have been demonstrated to have the capacity to reduce cerebral edema in animal models [23]. Their limited effect is partially explained by difficulties in passing BBB. Nowadays, new delivery methods like nanoparticle-based platforms or BBB-antibody conjugates are tested (Figure 1).

### 2.2. Seizures

Seizures are common symptoms in patients with primary BTs. Their incidence varies between 10% and 80% depending on tumor type [24] and is higher in patients with gliomas. Between 60% and 100% of low-grade glioma (LGG) patients present seizures, while in HGGs the seizure incidence is between 25% and 60%. In patients with diffuse glioma, seizures occur in about 50% of patients [25]. More than 25% of meningioma patients present seizures throughout their disease, one-third of them before first surgical intervention [25,26]. Regarding seizure type, in accordance with the International League against Epilepsy (ILAE), the most common types will now be referred to as ”focal” seizures and “focal to bilateral tonic-clonic” seizures [27], and one single seizure episode was diagnosed with epilepsy in patients with structural lesions like primary brain tumors [27]. Electroencephalography (EEG) is necessary in the initial assessment of patients with suspected seizures. It is also useful in determining future seizure risk; establishing a differential diagnosis; excluding nonconvulsive status epilepticus (NCSE), or diagnosing psychogenic seizures [2]. Seizure control can be realized with surgery, radiotherapy, and chemotherapy. In patients who have had one seizure, secondary prophylaxis with anticonvulsant is necessary. First-choice drugs should be levetiracetam and lamotrigine, although valproic acid has efficacy and is well tolerated. The guidelines indicate that enzyme-inducing antiepileptic drugs should be avoided in these patients [2]. In spite of treatment, one-third of these patients present seizure recurrence, which has a negative impact on the quality of their life [28]. Primary anticonvulsant therapy is ill-indicated in primary brain tumor patients [2].

Anticonvulsant therapy, which is useful for seizure control, is investigated in preclinical studies in order to determine the antiproliferative effects of these drugs. For instance, valproic acid had antiproliferative, antiapoptotic, and autophagic effects on human glioma cells [29,30]. Levetiracetam is another drug used for treating seizures in primary BT patients; its antiproliferative effects were tested on malignant glioma cells [31]. The same preclinical study demonstrated that the drug is capable of increasing malignant glioma cells’ sensibility to temozolomide and to inhibit O(6)-methylguanine-DNA methyltransferase (MGMT) [31]. Lacosamide also had a cytotoxic effect on human glioma cell lines [32] (Figure 1).

### 2.3. Stroke

Primary BT patients have one of the highest risks for fatal stroke among other cancer patients [33]. The risk of stroke in these patients is higher in the first 6 months and decreases 1 year after diagnosis [34].

Besides classic cerebrovascular risk factors like arterial hypertension, atrial fibrillation, diabetes mellitus, dyslipidemia, smoking, and so on, there are treatment-related risk factors and tumor-related factors. Among the tumor-related factors, the most important are tumor location, size, local vessel compression, vessel erosion, and autocrine factors (which can lead to hypercoagulability). The specific factors associated with primary BT treatment can be related to surgery (anesthesia, vessel injury), radiotherapy (microvascular changes in small and large vessels, aneurysms) and chemotherapy (temozolomide, which induces thrombocytopenia, cisplatin, bevacizumab). The analysis is based on an exceptionally large cohort of patients; it is a thought-provoking and great resource [35,36]. Actually, 25% of strokes in primary BT patients are a consequence of radiotherapy [37], while chemotherapy determines less than 1% of strokes [38].

Regarding symptomatology, any change in the neurological status of a primary BT patient needs evaluation (clinical and magnetic resonance imaging of the head for ischemic stroke or non-contrast computer tomography of the head for hemorrhagic stroke). Brain hemorrhage comes in different types, like epidural, subdural, subarachnoid, intraparenchymal, intratumoral, and intraventricular. A type of intraparenchymal bleeding is intratumoral hemorrhage, mostly encountered in high-grade tumors like GB. Usually, the patient also has thrombocytopenia or coagulopathies, or receives medications that increases the bleeding risk. The diagnosis of brain hemorrhage includes tests for coagulopathies [39].

Treatment of acute stroke in cancer patients is more restrictive compared with non-cancer patients. The presence of brain hemorrhage implies quick medical management. Besides general management, thrombocytopenia, if present, should be corrected with platelet transfusion. In patients receiving oral anticoagulants, rapid correction of elevated International Normalized Ratio (INR) is recommended by using fresh frozen plasma, vitamin K, prothrombin complex concentrates (PCC), activated Prothrombin Complex Concentrates-Factor Eight Inhibitor Bypassing Agent (PCC FEIBA), or recombinant activated factor VIIa.

Treatment of ischemic stroke in cancer patients is also more complicated when compared with non-cancer patients. There is little data regarding thrombolysis in patients with primary BTs. In primary BT patients, the administration of tPA is ill-advised because of intracranial hemorrhage risk [40]. On the other hand, in patients with benign primary BT and ischemic stroke, administration of tPA is considered safe [39] (Table 1 and Figure 1).

### 2.4. Other Cerebrovascular Complications—Moya-Moya Vasculopathy

Moya-Moya is a vasculopathy characterized by stenosis or occlusion of the distal intracranial segment of the internal carotid artery or the proximal anterior or middle cerebral artery associated with an abnormal collateral network at the brain base [41].

In a few cases the disease was described to coexist with primary BTs like GB, tuberculum sellae meningioma, ependymoma, pleomorphic xanthoastrocytoma, craniopharyngioma, cystic neurohypophysial germinoma, and pituitary adenoma [45,46,47,48]. In this situation, particular attention should be paid to surgery procedures for primary BTs, but also, the radiation dose and location should be adapted in order to avoid aggravation of Moya-Moya vasculopathy. Regarding chemotherapy administration, bevacizumab should be avoided for these patients [41] (Table 1 and Figure 1).

### 2.5. Neurocognitive Impairment

Neurocognitive impairment includes several significant declines in cognitive functions such as memory, attention, learning, calculus, executive functions (planning and decision-making), language, perceptual-motor skills, and social cognition, representing a drop when compared with a previous level. The patients present memory loss, confusion, diminished attention, disorientation, difficulty in performing familiar tasks, personality and behavioral changes, and aphasia. They should have a clinical assessment and neuropsychological testing along with imaging in order to establish the diagnosis [49].

Primary BTs cause neurocognitive dysfunction, with memory and executive functions being the most affected [50].

In these patients, the cognitive impairment is influenced by factors like tumor location, size, and histology, as well as the age of the patient and the treatment [51]. The transient or permanent cognitive deficit results from damaging the tumor-surrounding tissue [52].

Evidence suggests that the apolipoprotein E (ApoE) genotype influences neurocognitive decline in patients with BTs. In a clinical trial, Correa et al. found that patients with BTs with at least one ε4 allele exhibited significantly lower performances on tests of learning, memory, and executive functioning than those without the allele [53]. They also described other nine ApoE several single-nucleotide polymorphisms (SNPs) associated with attention, executive functioning, and memory [53]. The same authors demonstrated that in patients with BTs, cognitive domains like attention, executive functioning, and memory are influenced by other genotypes, catechol-O methyltransferase (COMT), brain-derived neurotrophic factor (BDNF), and dystrobrevin binding protein 1 (DTNBP1) [54]. In another study, Liu et al. investigated 10,967 SNPs mapping to 580 genes involving inflammatory, metabolism, and DNA repair pathways in glioma patients, and found associations with patients’ processing speed and executive functions [55]. They reported gene–gene interactions due to a linear relationship between the number of at-risk SNPs and the severity of neurocognitive decline. The authors also reported the association between DNA repair pathway genes and neurocognition: excision repair cross-complementation group 4 (ERCC4) rs1573638 was associated with processing speed, and the polymerase (DNA-directed) epsilon catalytic subunit (POLE) rs5744761 was associated with executive functioning. The associations between insulin receptor substrate and processing speed, as well as between executive function and nitric oxide synthase1 and interleukin-6, were also demonstrated [55].

The cognitive function of primary BT patients should be evaluated with neuropsychological tests like the Hopkins verbal learning test, the Reye Osterrieth complex figure test, the controlled oral word association test, the Stroop test, or the trail-making test. Self-perceived cognitive functioning reflects cognitive complaints experienced by patients and is assessed with validated questionnaires [49].

There are no guidelines for the management of neurocognitive impairment of BT patients. The treatment is usually done with donepezil, or memantine, or combination of methylphenidate and modafinil [42,43,44]. The cognitive deficit may be also reduced through cognitive rehabilitation, especially in young patients [56,57] (Table 1 and Figure 1).

## 3. Neurological Complications of Primary BT Treatment

### 3.1. Surgery

Although the complications after primary BT surgery are not very often reported, there is a classification first published in 2011 [58].

In a retrospective study published in 2025, 41.5% presented complications in the first 30 days after surgery; 10.7% were neurological complications. The limitations of this study include the small sample size, the retrospective nature, and the short follow-up period [59]. In another study published in 2022, out of 133 patients evaluated, 103 patients had a first surgery, and every fourth patient had complications after intervention. Among the postoperative neurological complications were bleeding into the ventricular system, hydrocephalus, postoperative hematoma, and brain edema. Actually, the post-surgery complications were present in between 9% and 40% of patients following BT surgery. The study limitations refer to the differences in group size, the fact that it is a single-center study and includes a relatively small number of patients, and the fact that the tumor sizes and precise locations were unknown [60].

All surgical complications in primary BT patients will provoke a delay in other treatment administration and will worsen patient prognosis. Among these neurological complications are sinus venous thrombosis, cerebrovascular events (ischemic or hemorrhagic), seizures, or hydrocephalus [3].

#### 3.1.1. Sinus Venous Thrombosis After BT Surgery

**Figure 2:**
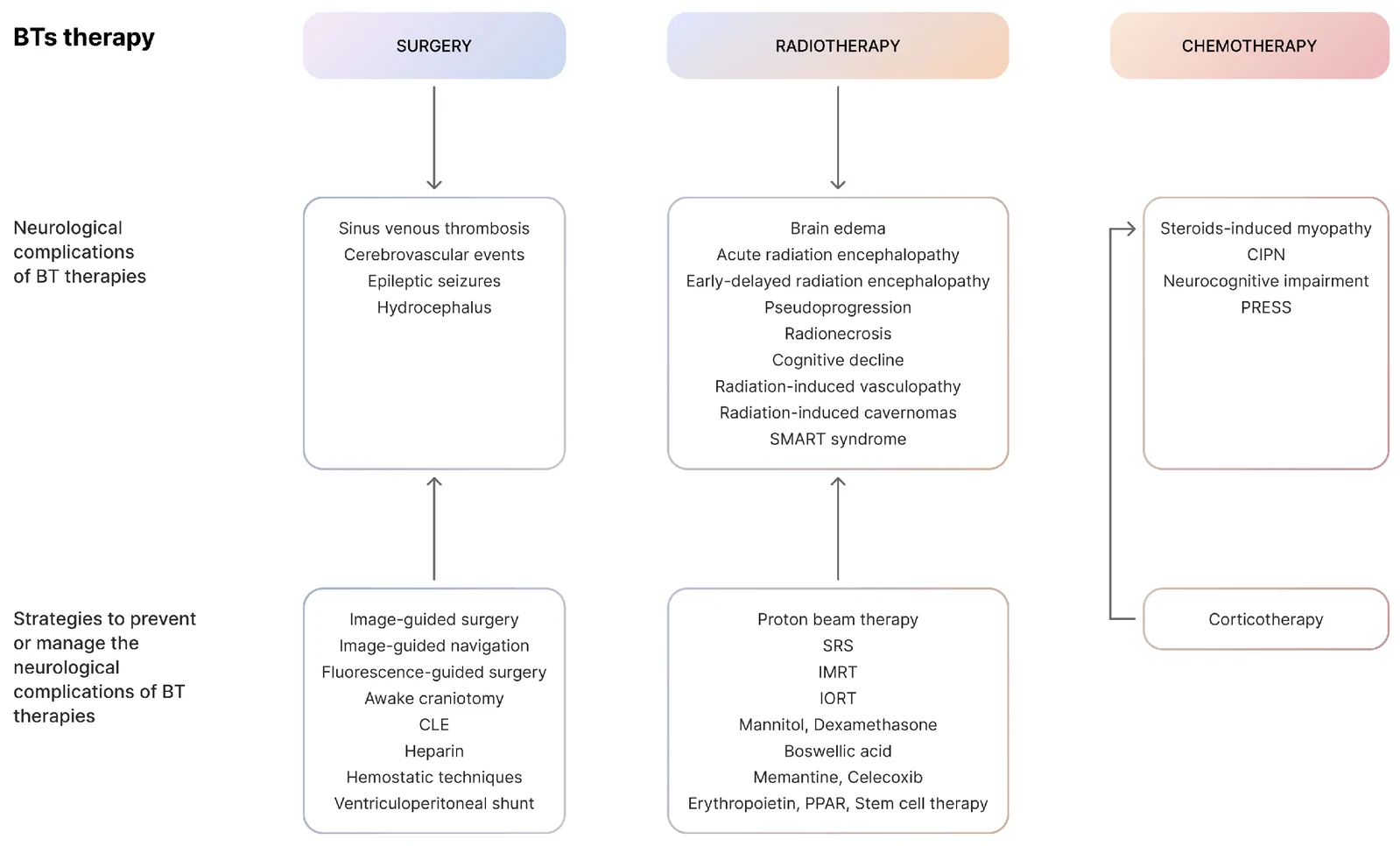
The major neurological complications of BT therapies and their relationship with the emerging therapies.

Sinus venous thrombosis after BT surgery is directly related with a surgical technique. It is a rare complication, a consequence of sinus injury after brain surgery. It represents about 5% up to 12% of cases, and it is mostly encountered after posterior fossa surgery [61]. Usually, this situation is incidentally found after postoperative imaging (either CT or MRI of the head). If the symptomatology is present, the complication should be suspected if the patient presents signs of intracranial hypertension, seizures, or a difficult recovery after anesthesia [62] (Figure 2).

#### 3.1.2. Cerebrovascular Events

Cerebrovascular events are important complications after primary BT neurosurgery. Brain ischemia is usually silent, discovered at routine imaging investigations. In one study published in 2024, Innes et al. reported that out of 244 patients with BTs (43% meningiomas and 21% GBs), 56% of diagnosed post-surgery strokes were ischemic; 38% of them were found in the first 30 days after brain surgery [63]. In malignant BTs, strokes caused greater functional loss and a higher mortality rate. Of these patients, 26% presented recurrent strokes. Being retrospective, the study had limitations [63]. In another study published in 2021, 44% of 539 patients operated on for diffuse gliomas presented postoperative infarctions, and there was a positive association between the infarction volume and tumor volume. The study was single-center and could have had false-positive findings as well, as the infarctions with hemorrhagic transformation could have been incorrectly scored [64]. These ischemic strokes may be a complication of surgery itself, but also the presence of coexisting medical conditions like atrial fibrillation are risk factors for these patients. The mechanisms of ischemic strokes following BT surgery are less studied; however, they are similar to those described for general ischemic strokes. The systemic inflammation and prothrombotic state may also be important in these patients [65]. There are also several pathogenic mechanisms that interplay between tumor proliferation and cerebral ischemia. Among them we can notice angiogenesis, but also the changes in the tumor microenvironment [66]. A higher risk of ischemic stroke was described in patients with repeated glioma resections [67].

There are no indications for thrombolysis for these patients [68].

Brain hemorrhage affected 44% of surgery-treated BT patients. The hemorrhages were more common in patients with malignant BTs; 35% of them were intratumoral. However, the study is limited by its retrospective design, and some patients may be non-responders to acetylsalicylic acid [63]. In another study published in 2024, the intracerebral hemorrhage in brain tumor patients who underwent surgery was 7.7% out of 650 patients [69]. Incomplete tumor resection is usually associated with higher incidence of neurological complications, like cerebrovascular events [70].

Regarding tumor type, postoperative bleeding after meningioma varies between 2% and 23% and it is influenced by factors like tumor location, size, vascularity, and so on [71,72]. In 2023, Ghare et al. considered that iatrogenic stroke and hematoma patients after brain tumor surgery have a 0-year incidence of 16.3/1000 and 10.3/1000, respectively [73]. There are several explanations, like excessive fibrinolysis, and therefore an increased predisposition for hemorrhage due to local tissue plasminogen activator; an increased hemorrhagic diathesis in tumors like vascular meningiomas; or GB driver mutations, tumor suppressors, and microRNAs, which dysregulate the coagulation system [74,75] (Figure 2).

#### 3.1.3. Epileptic Seizures

Epileptic seizures can occur either intraoperatively or post-surgery in patients with primary BTs. The intraoperative seizures appear especially during awake craniotomy, in patients known with epilepsy due to tumor presence and cortical mapping. The incidence of these seizures varies between 2.9% and 54.3% [76]. In glioma patients with cortical or subcortical stimulation and surgical manipulation, the intraoperative seizures are acute and considered symptomatic. One study in 2025 established that the presence of intraoperative seizures did not interfere with long-term seizure control. The same authors observed that early post-surgery seizures were present in 3% to 18% of glioma patients without previous epilepsy. In patients with intraoperative seizures, post-surgery epileptic seizures had higher incidence. The non-convulsive status epilepticus encountered intraoperatively was associated with higher morbidity and mortality in glioma patients. However, the study has limitations regarding its retrospective nature, and the series are not completely homogenous [77]. In another retrospective study, published in 2023, 3.5% of 200 patients had intraoperative seizures, and of them, 85.7% had a seizure history. Tumor location was associated with seizure incidence. The frontal lobe tumors were connected with a higher risk of intraoperative seizures. The study is limited by the small sample of patients; also, the use of pre-operative antiepileptic drugs could not be reported, and the previous predictors like tumor volume, tumor margins, or IDH1 status could not be studied [78]. Another retrospective study, also published in 2023, showed that out of 113 meningioma patients who were treated with surgery, 9.7% presented postoperative seizures without having a previous epilepsy diagnosis. The convexity meningiomas, as well as tumor size, were most frequently involved. This study was also retrospective and therefore had limitations [79]. Regarding prophylaxis, the guidelines do not recommend prescribing antiepileptic drugs as primary prophylaxis in the peri- or postoperative situation, even for awake surgery [80] (Figure 2).

#### 3.1.4. Hydrocephalus

Hydrocephalus is an abnormal accumulation of cerebrospinal fluid in the cranial cavity. The collection is mainly in the ventricular system. BT patients can have either obstructive or communicating hydrocephalus. After BT surgery, the incidence of hydrocephalus can be from 7.3% up to 46% of cases. In one study published in 2024, 25.1% out of 179 BT patients developed hydrocephalus after surgery and needed a shunt. The postoperative hydrocephalus was associated with glial tumors, intraventricular extension of the tumor, intraventricular hemorrhage, and residual tumor volume. Also, patients with recurrent BTs have a greater risk of developing this pathology. Patients with tumors of midline structures have a higher risk of post-surgery shunting. The major limitations of the study were heterogeneities in patients’ age, tumor location, and pathology [81]. Regarding GB, one study published in 2025 reported that out of 781 operated patients, 3.6% developed hydrocephalus. Of these, 54% were treated either with ventriculoperitoneal shunt or endoscopic procedures, with the improvement of symptoms in 88% of patients [82]. After meningioma surgery, the incidence of postoperative hydrocephalus varied between 13.3% and 16.6% [83] (Table 2 and Figure 2).

### 3.2. Radiotherapy

Usually, patients with primary BTs receive conventionally fractioned radiotherapy to partial brain. Radiation therapy plays a crucial role in the management of these tumors. The neurological side effects of this type of therapy are either acute or chronic. The mechanisms responsible for this reaction is even nowadays incompletely understood. There are several pathogenic mechanisms capable of explaining these processes: a neurogenesis reduction, cellular microenvironment changes, oxidative stress, endothelial cell death, accelerated atherosclerosis, and microangiopathy [84].

During brain irradiation, there is an infiltration of the brain with immune cells capable of producing reactive oxygen species (ROS). Oxidative stress is a consequence of the imbalance between the production and removal of ROS [85]. This process initiates cell death, and as a consequence, ROS contributes to neural toxicity.

Another pathogenic mechanism is nonspecific inflammation which causes endothelial cell apoptosis and BBB break-down, hypoxia, and peritumoral edema [86]. Also, radiation therapy can induce astrocyte proliferation, stimulating inflammation, but also possibly activating microglias [87]. Radiation also can induce changes in vascular permeability which are dependent on cyclooxygenase 2 (COX2) [88]. The transient demyelination after radiotherapy can be explained by the loss of oligodendrocyte type-2 astrocyte (O-2A) progenitor cells. The vascular damage produced by irradiation can also be a consequence of oxidative stress and neuroinflammation [89].

Acute toxicities are present less than 90 days from the beginning of treatment. They are usually transient and include brain edema, the worsening of neurological deficit, seizures, acute radiation encephalopathy, and brainstem syndrome [90] (Figure 2).

#### 3.2.1. Brain Edema After Radiotherapy

Brain edema after radiation therapy of BTs is influenced by the dose of radiation, the volume irradiated, mainly in regions as thalamus or corticospinal tracts, or the administration of chemotherapy at the same time [91,92]. It is a result of a transient increase in vascular permeability, a consequence of the vascular injury provoked by radiation. Because irradiation exacerbates the swelling around the tumor, it aggravates symptoms like nausea, headaches, vomiting, nuchal rigidity, motor and sensory deficits, and seizures [93]. However, the exact pathophysiology of postirradiation brain edema is still unclear. The process can be related to damage to microglia and astrocytes, disruption of the BBB, or cerebrovascular impairment.

For instance, regarding meningiomas, brain edema after radiotherapy was found in about 5.5% out of 293 patients, as Aissaoui et al. reported in 2025 [94]. The median time to onset was 3 months and the highest rate was in midline meningiomas (13%). Among the predictive factors for post-radiotherapy brain edema were tumor size, midline location, and grade 2 histology. The study has limitations, such as its retrospective design, heterogeneous population, and potential selection bias [94] (Figure 2).

#### 3.2.2. Acute Radiation Encephalopathy

Acute radiation encephalopathy appears in the first 2 weeks after the first irradiation, and it presents with nausea, vomiting, dizziness, headache, and the worsening of the already existing neurological deficit. Usually, this pathology is associated with high doses of radiotherapy and larger tumors. The mechanism is not well described; however, a BBB disruption accounting for a rise in intracranial pressure seems to explain the symptoms [86]. Nowadays, with the use of lower doses of fractioned whole-brain radiotherapy, this complication almost never exists. The treatment is surgical removal of the tumor, and where this is not possible, corticotherapy with dexamethasone is used [95]. However, in 2025, Liang et al. presented a study which meant to realize a prediction model for acute radiation-induced brain injury in patients with glioma [96]. It was a retrospective study, which presented data from 420 glioma patients. They observed that age, tumor volume, and chemotherapy administration were independent risk factors for radiation-induced brain injury in these patients. However, the study was limited because it was a single-center retrospective clinical study [96] (Figure 2).

#### 3.2.3. Early-Delayed Radiation Encephalopathy

Early-delayed radiation encephalopathy is a condition characterized by subcortical frontal dementia, gait apraxia, and urinary incontinence. There are several mechanisms involved in the pathophysiology of the disease, such as a transient demyelination or oligodendroglial injury. The diagnosis is established using symptoms, examination, MRI, and EEG. Corticoids associated with radiotherapy and after treatment are capable to help [93].

In 2021 were published the results of a retrospective study of 1391 patients with HGGs, of whom 44 presented radiation-induced leukoencephalopathy. The risk correlated with age (over 60), radiation volume dose, and tumor location. Also, the authors observed that maybe there were individual factors affecting brain sensitivity to radiation, because the white matter changes were progressive and involved both hemispheres [97] (Figure 2).

#### 3.2.4. Pseudoprogression

Pseudoprogression is a worsening of the neurological deficits that patients with primary BTs have already presented before irradiation, and usually occurs in the first 12 weeks after radiotherapy cessation. It is a consequence of an increase in tumor volume of at least 25%, which resolves without a cancer treatment change, but only with steroids [98]. It can be found in about 30% of patients; the MRI may present with signs of tumor progression, but after a few months, the images improve without treatment. Actually, it is a challenge to distinguish between tumor progression and pseudoprogression. Advanced magnetic resonance imaging (MRI) techniques such as diffusion-weighted imaging, perfusion MRI, and proton magnetic resonance spectroscopic imaging are more efficient than conventional MRI in differentiating pseudoprogression from progression. Nowadays, computer science and artificial intelligence (AI) present new possibilities in the imaging field with radiomics [99].

Pseudoprogression is usually encountered in GB patients treated concomitantly with alkylating agents like temozolomide. Blakstad et al. presented in 2024 a retrospective study in which 284 patients diagnosed with GBs and treated concomitantly with radiotherapy and temozolomide were followed up with MRI [100]. The incidence of pseudoprogression at 3 months after irradiation was 19%, while at 6 months after treatment, it was 7%. The study is limited by the known biases inherent to retrospective analysis [100]. The overall progression rate was 21.5% higher than the previous AVAglio trial published in 2014 [101]. In 2022, Stock et al. published the results of a multicentric study which followed 136 patients diagnosed with LGGs treated with radiotherapy [102]. Of these patients, 39.7% presented pseudoprogression. The study was retrospective. In 2022, Maksoud et al. reported a case of meningiomas treated with stereotactic radiotherapy [103]. Pseudoprogression was observed in 6% of patients. The study had limitations because it was retrospective, the number of cases was small, and there was a lack of information regarding the toxicity of the treatment [103] (Figure 2).

#### 3.2.5. Radionecrosis

Nowadays, radionecrosis is a rare complication of brain radiation therapy. In patients receiving 1.8–2.0 Gy per fraction less than 3% present severe radionecrosis. In re-irradiated patients the risk increases by 5% up to 20% [104]. There are several factors that influence this complication, like radiation dose, fractionation, prior radiation, other therapies, tumor biology, advanced age, and underlying vascular disorders [105]. Although several pathophysiological mechanisms are discussed, radiation necrosis development is not yet fully understood. The presence of edema and of tumor render parenchyma in the tumor bed, representing a susceptibility to radionecrosis. Radiation therapy ionized oxygen species that interact with the cellular DNA and diminish cells capacity of DNA repair. In the end there was a process of apoptosis of the tumor cells [106]. The differentiation between tumor recurrence and radiation necrosis remains a challenge in spite of advances in imaging investigations [107]. The clinical picture is represented by various neurological changes, from focal deficits to headache, cognitive decline, or seizures [108].

Regarding gliomas, radiation necrosis occurs in 9.8% up to 44.4% of patients [109]. In one retrospective study published in 2025, of 257 patients with GB, 95 were treated in accordance with Radiation Therapy Oncology Group guidelines and had a higher rate of radiation necrosis, at 34%. On the contrary, the 162 patients treated in accordance with MD Anderson Cancer Center guidelines had an incidence of radionecrosis of 21% [110]. In another retrospective study published in 2023, of 110 GB patients with a second resection, 21% had radionecrosis. The limitations of the study are represented by the retrospective nature; also, data on toxicity grading were retrospectively assigned; radionecrosis included patients who were both asymptomatic and symptomatic; radionecrosis may have been a mixture of viable tumor and treatment effects; and data regarding patients’ cognition or quality of life following radio therapy and detailed dosimetric volumes were not collected [111].

The incidence of radionecrosis after radiation therapy for meningioma patients varies between 0.1% and 23% [112]. One retrospective study published in 2026, reported that out of 473 patients with intracranial meningioma, only 4% developed radiation necrosis. The risk was higher for larger tumors [113]. In another retrospective study published in 2025, out of 135 patients diagnosed with low-grade meningiomas who received conventionally fractionated radiotherapy, 15% developed radiation necrosis. The study had limitations: it was retrospective and single-center, and pathological confirmation and advanced brain MRI techniques were not required for a diagnosis [114] (Figure 2).

#### 3.2.6. Cognitive Decline

Among the late effects of BT radiation therapy are the neurocognitive changes. They are thought to be irreversible and with a progressive evolution. The cognitive changes in these patients include deficits in attention, memory, information processing, and executive function [115]. The pathophysiological mechanisms of the neurocognitive decline of primary BT patients after radiation therapy is poorly understood. The increased risk of neurocognitive decline is suggested as being a consequence of the radiation dose to the hippocampus or cerebellum [116,117]. One retrospective study published in 2024 presents the situation of 219 patients diagnosed with primary BTs and treated with radical photon and/or proton radiotherapy (RT). Up to 50% of patients developed neurocognitive decline. The study had limitations because it was single-centered and had a limited follow-up period [118]. Another retrospective study published in 2024 investigated the impact of high-dose radiotherapy on cognitive impairment of 72 BT patients. High-dose radiotherapy could aggravate cognitive impairment. However, there are limitations of this study. There was only a small sample, a lack of chemotherapy information, comorbidities in the patients, and an absence of information regarding the brain area and volume that received radiotherapy [119]. The severity of the cognitive decline induced by radiation therapy is dependent on the age at the irradiation in primary BT patients [120]. The result of eight studies which included 310 primary BT patients was that proton therapy, capable of sparing tumor-surrounding healthy tissue, seems to spare cognitive function [121]. There is also a correlation between the cognitive status and the dose of radiation of several brain regions. For instance, irradiation of the left hemisphere, hippocampus, or prefrontal regions is strongly connected with the cognitive status [122] (Table 3 and Figure 2).

#### 3.2.7. Radiation-Induced Vasculopathy

Another complication of brain radiotherapy is the vasculopathy of large or small arteries, which is an accelerated form of atherosclerosis and generally occurs years after irradiation. Among the affected intracranial arteries is the distal internal carotid artery. It can present stenoses or even occlusions as a consequence of radiation therapy. Cerebral arteries can be affected as well [130]. The period between the exposure to radiation and vasculopathy development varies between 2 and 20 years.

Pathophysiological mechanisms are complex and involve endothelial cells, reactive oxygen species, and cytokines, but also angiogenesis and new blood vessel formation. Radiation will induce endothelial cell injury and vascular inflammatory processes, both depending of the dose and duration of exposure to radiation [131].

Regarding the symptomatology, patients will present focal ischemia or hemorrhages, headache, vertigo, impaired consciousness, and seizures [132]. One retrospective study published in 2024 presented the incidence of ischemic and hemorrhagic strokes in 116 patients diagnosed with primary BTs receiving irradiation compared with 85 non-irradiated primary BT patients. The patients who received radiation therapy had a longer survival had an increased stroke prevalence. The study had limitations like the unknown tumor contouring and a chronological bias [133]. In 2020, another retrospective study presented the increased risk of early cerebrovascular disease and white matter hyperintensities in 70 child BT survivors. Most lesions were located in the high-dose radiated brain area. The study was limited by its cross-sectional design and the lack of follow-up regarding vascular changes. The differences in chemotherapy protocols and radiotherapy techniques were other limitations [134]. It is also known that the survivors of childhood BTs need screening for vasculopathy after receiving radiotherapy, especially with higher doses [131]. Actually, children with vasculopathy induced by irradiation have a higher risk of developing an ischemic stroke (a 5-year recurrence of 66%) [127] (Table 3).

Moya-Moya disease is a progressive occlusive cranial vasculopathy, characterized by abnormal anastomoses and narrowing of intracerebral blood vessels. The blood flow is diminished and therefore the risk of ischemic stroke, transient ischemic attacks, or seizures is increased. BT irradiation is a contributing factor to Moya-Moya syndrome, especially in patients who are children or younger patients at the time of radiation therapy [135,136]. If a young patient has neurofibromatosis type 1, there is the indication to replace radiotherapy with chemotherapy because of the increased risk of developing Moya-Moya disease after irradiation [137] (Figure 2).

#### 3.2.8. Radiation-Induced Cavernomas

These are rare complications of high-dose radiation therapy, especially in young patients diagnosed with BTs. These cavernomas usually develop in previously high-dose-irradiated areas of the brain with a mean latency of one decade, but also at the margins of the radiated field or at distant sites like the frontal or temporal lobes [128]. The mechanisms involved are not certain; however, higher expression of hypoxia-inducible factor 1 (HIF-1) and vascular endothelial growth factor (VEGF) seem to increase the susceptibility of BT patients, especially young ones, in developing cavernomas years after receiving radiation therapy [138]. Patients with LGGs, especially children, who need high-dose radiation therapy more frequently exhibit cavernomas after treatment. There are several factors that influence the development of cavernomas after primary BT irradiation, such as the radiation dose, no matter the radiation type (standard photon therapy, proton therapy, or gamma knife surgery), or cerebral cavernous malformation (CCM) gene mutations [139] (Table 3). A number of recent systematic reviews conclude that the long-term follow-up of these patients is very important [128,139] (Figure 2).

#### 3.2.9. SMART Syndrome

Another rare late complication of BT radiation therapy is SMART syndrome. Patients complain of migraine-like headaches, seizures that respond poorly to anticonvulsants, and focal neurological deficits. A pial enhancement and a cortical high signal change can be found when patients are investigated with imaging. Pathophysiological mechanisms like vascular dysfunction, chronic inflammation, or blood–brain barrier disruption of this syndrome are still discussed. In 2025, Relloso de la Fuente et al. published a series of cases—patients with primary BTs who presented with SMART syndrome after irradiation [140]. Similar clinical cases were presented in 2023 by Dossin et al. [141], Duranikova et al. [142], and Chow et al. [129]. Regarding the treatment, corticosteroids are inefficient and there is no actual treatment for this syndrome (Table 3) (Figure 2).

### 3.3. Chemotherapy

As with all cancer survivors, brain tumor patients can present neurological complications after exposure to anticancer therapy, including chemotherapy.

#### 3.3.1. Steroid-Induced Myopathy

Used to reduce brain tumor-surrounding edema, steroids remain an important tool in releasing symptoms like headache, nausea, or focal neurological deficits. One neurological side effect of continuous administration of steroid treatment is myopathy. Elderly patients have a higher risk of developing the disease because of lower muscle mass. Other risk factors are prior muscle disease, spinal cord injury, chronic respiratory illness, poor nutritional status, or a sedentary lifestyle. Women are more affected than men [143]. The pathogenic mechanisms of the disease are both catabolic and anabolic. Corticosteroids are capable of upregulating the proteolytic systems like calpains, cathepsins, or the ubiquitin-proteasome system, but also can induce myocyte apoptosis. Also, corticoids inhibit amino acid transport into the cells [144].

Muscle weakness is symmetrically distributed in the proximal muscles of the extremities, and the hip girdle is affected earlier than shoulders. Usually, muscle weakness is associated with myopathy may also worsen other neurological deficits such as motor deficit or ataxia. The onset of the disease is usually month after corticoid therapy initiation. Patients can climb stairs or perform overhead activities with difficulty. At the neurological examination of muscle weakness, decreased muscle stretch reflexes in the affected extremities, or sensory deficits, can be found [145]. Laboratory investigations are represented by creatine kinase, aspartate aminotransferase, lactate dehydrogenase, and aldolase [146] (Figure 2).

Steroid-induced myopathy in patients with BTs has a negative impact on their quality of life [147].

#### 3.3.2. Chemotherapy-Induced Peripheral Neurotoxicity (CIPN)

CPIN is a late adverse event that can be encountered in patients diagnosed with BTs and treated with vinca alkaloids or oncogenic fusions of the neurotrophic tyrosine receptor kinase inhibitors (NTRKIs).

CPIN is as a length-dependent sensory axonal polyneuropathy. The usual clinical signs are numbness/paresthesia and/or neuropathic pain in a stocking-and-glove distribution. Patients also present changes in proprioceptive sensation and sensory ataxia, which can increase the risk of fall. Impaired strength is usually mild and distally encountered. The evolution of the disease is influenced by chemotherapy administration in a dose-dependent and cumulative manner [148].

The detection, monitoring and grading of CPIN are still discussed. It seems that the best way to evaluate CIPN is a combination of clinician-reported outcomes and patient-reported outcomes. Several grading scales, like the Total Neuropathy Score or TNSc (TNS clinical), are used. Also very important are nerve conduction studies (NCSs) [149]. The testing of the most distal branches of the peripheral nervous system, such as the dorsal sural nerve, is particularly useful. Also, imaging techniques like ultrasound and magnetic resonance neurography can be used for disease monitoring. Among the biomarkers that can be used, neurofilament testing is the most promising [150,151].

Patients with BTs usually receive a combination of vincristine with procarbazine and lomustine in the PCV regimen. Vincristine is a component of vinca alkaloids with a poor oral bioavailability. Therefore, it is usually administrated intravenously (i.v.). The drug has a poor BBB penetration, but is able to reach the contrast-enhancing tumor areas in the CNS known for BBB leak. Vincristine is metabolized in the liver and the half-life is about 80–85 h [152]. In PCV regimen, the drug dosage is 2 mg on days 8 and 29 [153]. Axonopathies leading to axonal sensorimotor neuropathies of large and small nerve fibers are the main complication of the drug administration. The clinical signs are numbness, paresthesia, and/or neuropathic pain in a stocking-and-glove distribution, which usually evolves during chemotherapy in a dose-dependent, cumulative manner. At the neurological examination, patients present with distal, symmetric, upper and lower limb impairment, or even loss of all sensory modalities, and distal, symmetric weakness in the lower limbs, progressing to foot drop. Deep tendon reflexes are reduced or lost [154].

In spite of chemotherapy’s low penetration of the BBB, cranial nerve neuropathies have been described, mainly in children. The patients presented various cranial nerve clinical signs, like ptosis, ophthalmoplegia, diplopia, strabismus, ocular muscle paresis, color vision deficiency, blindness, blurred vision, limited mobility of jaw and facial muscles, hearing loss, reduced tongue movements, and stridor [155].

Regarding the pathophysiology of the polyneuropathy, vincristine can target microtubules blocking cancer cells in metaphase, promote microtubule depolymerization, alter axonal transport, or produce alterations in mitochondrial fission/fusion processes [156]. In the past decade, a few other mechanisms have been studied and discussed, such as the sterile and Toll/interleukin 1 receptor (TIR) motif containing protein 1 (SARM1) and the potential involvement of neuroinflammation in axonal damage [157,158].

In clinical trials, although little data is available, the few retrospective studies report that the drug neurotoxic effects on primary BTs was significant. There were limitations of the studies: certain elements of the studies were conducted retrospectively, and the studies were of a subjective nature [159,160].

Larotrectinib is a first-generation NTRK inhibitor and approved by the FDA and EMA since 2018 for TRK fusion-positive cancers [161]. Initially used as an orphan drug for soft tissue sarcoma, after positive results in clinical trials published in 2017, the drug is now approved to treat any cancer containing mutations, as opposed to cancer-specific tissues. It is the first available oral, highly selective TRK kinase (TrkA/B/C) inhibitor approved for the treatment of site-independent tumors in adults and children with NTRK gene fusions [162]. In BT patients, the drug had positive results in patients with TRK fusion-driven HGGs and LGGs [163,164,165,166]. The recommended dose in adults is 100 mg larotrectinib twice daily. In pediatric patients, it is 100 mg/m^2^ larotrectinib twice daily, with a maximum of 100 mg per dose. From birth to less than 3 months, the recommended starting dose is 50 mg/m^2^ twice daily [167]. Besides other neurological adverse events like dysgeusia and dizziness, the drug provokes peripheral sensitive neuropathy. However, the studies had limitations: the histologic review was performed locally without central review, detailed methylation data were limited, and radiological assessments for patients with primary CNS disease were investigator-assessed and collated locally at each study site [168,169]. Nowadays, there are ongoing clinical studies using larotrectinib for newly diagnosed patients with HGGs with NTRK fusion [170].

Entrectinib is also a first-generation NTRK inhibitor approved by the FDA in 2019 for treatment of solid tumors with NTRK fusions [171]. The dosage of the drug is 600 mg orally once a day. In pediatric patients, the dosage varies between 400 mg and 600 mg daily [167]. Regarding BTs, the drug was mainly studied on pediatric patients in the open-label clinical trials STARTRK-NG and TAPISTRY [172]. Of 33 patients, 17 had primary tumors of the central nervous system. Entrectinib induces rapid and durable responses in children with NTRK or ROS1 fusion-positive tumors. Besides other neurological adverse effects like dizziness, balance disorder, taste disorder, ataxia, cognitive disorder, memory impairment, aphasia, dyslalia or dysarthria, the drug can induce peripheral sensitive neuropathies. The study had limitations stemming from the use of spontaneous reporting data [168,173,174] (Table 4 and Figure 2).

Regarding the treatment of these neuropathies, currently no preventive or curative therapy is available.

#### 3.3.3. Neurocognitive Impairment

Cognitive decline in patients with BTs is common both in treated and untreated patients. Patients present impairment of several cognitive domains like memory, attention, and language, which can affect the quality of life and overall survival. Among chemotherapies used for brain tumor treatment, temozolomide is a standard chemotherapeutic agent used for GB patients. Temozolomide targets nuclear DNA and generates nuclear DNA adducts that induce cell cycle arrest and cell death by apoptosis. The drug also alters mtDNA and respiratory function in glioma [183]. When administrated with concomitant radiotherapy, the dosage is 75 mg/m^2^ [184]. Patients without radiotherapy receive 150 mg/m^2^ up to 200 mg/m^2^ daily [185]. The drug is capable of causing neurotoxicity and cognitive decline, including impairment of executive function and memory, which are correlated with dosage [175,176,186]. Combined therapies are capable of improving survival outcome but are also able to increase cognitive decline [177].

Bevacizumab, a monoclonal antibody, is another chemotherapy drug used in GB therapy which is generally well tolerated. In brain tumor patients, it is i.v. administered at 5–15 mg/kg every 2 weeks [187]. Neurogenesis plays an important role in structural plasticity and network maintenance, which is essential for intact cognitive function. The altered neurogenesis is an early critical event in the course of severe cognitive decline. Bevacizumab treatment impairs microstructure in mesiotemporal (hippocampal) regions and in frontal regions [188]. There are studies that report cognitive decline in patients treated with bevacizumab [178,179]. One explanation is that VEGF promotes neurogenesis, with an important role in structural plasticity and network maintenance, both essential for cognitive function [189] (Table 4). Regorafenib is another chemotherapy drug used since 2020 for GB treatment. The dosage is 160 mg orally, every day for 3 weeks [190].

Its effects on cognitive function of patients with GB were also studied in [180] (Table 4).

Nitroureas are cytotoxic chemotherapy drugs that cause cell death by DNA damage, used to treat GBs. Nitrosourea compounds have led to usage in animal experiments to induce neurotoxicity and simulate Alzheimer disease [181]. The dosage used for BT patients was 150 mg/m^2^ [191] (Table 4 and Figure 2).

Unfortunately, there are no specific measures to improve this condition.

#### 3.3.4. PRES

PRES is a rare neurologic disorder characterized by seizures, altered mental status, headache, visual disturbances or cortical blindness. The patients may or may not exhibit arterial hypertension. The diagnosis of this disorder is based on clinical and MRI findings. Regarding the pathophysiology, several theories, like the impairment of cerebral autoregulation or endothelial compromise, are discussed [192]. A chemotherapy drug used for GB treatment reported to induce PRES in isolated patients, is bevacizumab is a VEGF inhibitor [182] (Table 4 and Figure 2).

## 4. Current Strategies to Prevent or Manage the Neurological Complications of BT Treatment

### 4.1. Strategies to Prevent or Manage Surgical Complications

Postoperative complications in patients with BTs delay chemotherapy administration, and are linked with worse survival outcomes, especially in GB patients. An important problem in BT therapy, especially GB, is finding the equilibrium between maximum tumor removal and preservation of neurological functions. Although an aggressive tumor resection may improve patient outcome, the possibility of postoperative neurological complications is increased. Therefore, prevention of these neurosurgical complications becomes crucial for these patients [4]. Advanced neurosurgical techniques are used nowadays during BT resection help to prevent these complications. Among them, advanced mapping methods and awake craniotomy help neurosurgeons to achieve the maximum tumor resection and preserve the neurological function without neurological complications. The use of individualized surgical techniques, intraoperative neuromonitoring, with the aid of several specialists realizes this fragile balance [193].

Another important problem during BT surgery is the achieving of maximal gross resection. This is particularly difficult when the BT is next to brain regions responsible for important neurological functions. In this situation the preservation of neurological functions by using intraoperative mapping is the answer for these patients [194].

#### 4.1.1. Image-Guided Surgery

Nowadays, there are various imaging techniques that allow neurosurgeons to better visualize BT boundaries and minimize the damage to healthy tissue. Intraoperative techniques like intraoperative magnetic resonance imaging, intraoperative ultrasound, or confocal assisted multispectral fluorescent microscopy help the neurosurgeon during intervention by providing real-time images.

Intraoperative magnetic resonance imaging provides real-time imaging during surgery, enhancing the safety of BT resection [195]. With its help, the neurosurgeon can better visualize tumor boundaries and better resect the infiltrative areas difficult to distinguish from normal brain tissue, optimizing the extent of tumor resection. It also helps by minimizing the postoperative neurological deficits, because of a better preservation of critical brain structures [195]. A meta-analysis published in 2025 investigated the efficacy of this imaging technique used during glioma neurosurgery. In 29.2% of cases, extended resections were performed with intraoperative magnetic resonance imaging guidance, achieving the gross total resection. These patients had a median time to progression of 16 months. Unfortunately, the neurological deterioration persisted. The limitations of the study are the lack of molecular data, low statistical power, and the difficulty in drawing definitive conclusions [196]. Other recent studies, performed in the last few years, confirm that the technique reduces residual tumor volume in GB patients, but they also have limitations regarding selection bias or molecular analysis [197,198].

Intraoperative ultrasound uses high-frequency sound waves that are emitted and detected by a handheld probe. The method provides real-time high-resolution images of the BT, allowing neurosurgeons to better identify tumor boundaries and the extent of tumor resection. It is an alternative to intraoperative magnetic resonance imaging [199]. Altough it does not improve progression-free survival, the technique helps surgeons to achieve gross tumor resection for HGG and GB patients [200]. Other studies suggest that B-mode ultrasound helps neurosurgeons to completely resect HGGs or at least have a smaller residual tumor. One limitation of the study was that it was not double-blinded and only 2-D B-mode intraoperative ultrasound imaging was used [201]. Similar results were obtained for glioblastoma patients [202]. When combined with navigation technology, intraoperative ultrasound achieves similar results to intraoperative magnetic resonance imaging regarding gross tumor resection rate, with lower costs [203]. Although mainly used for glioblastoma surgery, image-guided surgery proved its efficacy for meningioma treatment, especially deep tumors, reducing the post-surgery neurological complications [204] (Figure 2).

#### 4.1.2. Image-Guided Navigation

Image-guided navigation uses computer-assisted navigation systems capable of creating a 3-D map of the patient’s brain by using preoperative imaging data. The technology helps neurosurgeons to better locate and remove tumors, especially those located in deep areas of the brain. For instance, mixed-reality guidance used for GB resection improves the quality of tumor resection, diminishing the neurosurgical complications [205]. Also, intra-operative navigation can be optimized through augmented reality for glioma surgery and improve the functional outcome of these patients [206] (Figure 2).

#### 4.1.3. Fluorescence-Guided Surgery

Fluorescence-guided surgery using 5-aminolevulinic acid (5-ALA) is a neurosurgical technique capable of helping surgeons perform better resections of GB tissue. This is the consequence of the accumulation of 5-ALA in GB cells and determining them to emit a red fluorescence at a specific wavelength of light. 5-ALA is administrated to GB patients prior to surgery, and allows surgeons to differentiate between tumor tissue and normal brain tissue. With this technique the rate of gross tumor rection is improved, as well as patients’ outcomes. One study published in 2026 using fluorescence-guided surgery for HGG patients reported improved short- and medium-term survival outcomes without an increased risk of postoperative neurological morbidity. The limitations were that the study was retrospective and not randomized, and it lacked molecular profiling [207,208]. Fluorescence-guided surgery is also used in evaluating the extent of resection, especially in patients with complex meningiomas. The method is mainly used for recurrent meningiomas and for irradiated meningiomas [209] (Figure 2).

#### 4.1.4. Awake Craniotomy

The protection of vital functions along with maximum tumor resection is the most important goal of surgeons while operating on BTs. One approach that helps neurosurgeons achieve these goals, along with the reduction of neurological complications after surgery, is the use of awake craniotomy with intraoperative mapping. This technique uses real-time mapping, monitoring the brain functions while the patient is awake, thus reducing the postoperative neurological deficits and complications. However, the studies had limitations, being single-arm and retrospective [210,211]. Awake craniotomy is also considered for surgical resection of convexity and parasagittal meningioma [212] (Figure 2).

#### 4.1.5. Confocal Laser Endomicroscopy (CLE)

CLE is a method which permits the visualization of very small structures and hidden anatomical details. It is used for BT surgery. Nowadays its effectiveness is augmented by the use of AI-based diagnostic models [213] (Figure 2).

#### 4.1.6. Strategies to Prevent or Manage BT Surgery Sinus Venous Thrombosis

The gold-standard treatment is heparin. After 15 days, patients will switch from heparin to oral anticoagulation for about 3 months, unless thrombophilia is diagnosed and long-term oral anticoagulation is needed [60]. Although most patients have a good prognosis, they may continue to have neurological symptoms like headaches, cognitive impairment, seizures, and vision loss [59] (Table 2). In refractory cases, endovascular therapy should be taken into account. Regarding prevention, adequate hydration and minimizing sinus injury are recommended. Also, prophylactic anticoagulation after surgery, mainly in patients with risk factors, should be considered [61] (Figure 2).

#### 4.1.7. Strategies to Prevent or Manage Cerebrovascular Events After BT Surgery

For patients who suffer ischemic stroke after BT surgery, there are no indications for thrombolysis [68]. In order to prevent hemorrhage after BT surgery, there are hemostatic techniques like mechanical hemostasis through tamponade with small gauze wads, thermal hemostasis utilizing electrocautery, and local hemostatic agents (gelatin sponges, fibrin glue, other active flowable hemostatic substances). Also, monitoring of the coagulation status of the patient is important. If the patient presents coagulation disturbances, targeted therapy with coagulation factors and procoagulant medication is indicated [214] (Figure 2).

#### 4.1.8. Strategies to Prevent or Manage Epileptic Seizures After BT Surgery

The prophylaxis of epileptic seizures after BT surgery is not recommended. The guidelines do not recommend prescribing antiepileptic drugs as primary prophylaxis in the peri- or postoperative situation, even for awake surgery [80]. Regarding the treatment, first in line is levetiracetam, but valproate is also effective for GB patients and has potential survival benefits. However, the studies has limitations the lack of information on seizure frequency, missing neuropathological data, and a small patient group [215,216]. If one drug cannot control seizure frequency, the supplementation of the dose and the aid of another drug are the indicated choices (Figure 2).

#### 4.1.9. Strategies to Prevent or Manage Hydrocephalus After BT Surgery

Ventriculoperitoneal shunt placement is the most frequently utilized method for hydrocephalus treatment. In patients with limited survival prognosis, endoscopic ventriculostomy helps to avoid the complications related with shunt placement [217]. Regarding prevention, the use of predictive scores for predicting the chance of development of postoperative hydrocephalus is useful. The scores also help to identify patients who need closed clinical and radiographic surveillances of postoperative hydrocephalus [81] (Figure 2).

### 4.2. Strategies to Prevent or Manage Radiation Therapy Complications

Radiotherapy is another essential tool in controlling tumor growth, although, unfortunately, it may induce acute and long-term neurological complications. While targeting tumor cells, radiation therapy may also damage healthy brain structures. For instance, when white matter tracts are affected, the patient will be confronted with long-term neurological deficits. When the hippocampus is affected, the patient will present neurocognitive deficits. The focus nowadays is to preserve the healthy neural structures and function and consequently improve BT patients’ quality of life [218]. The use of modern methods of irradiation rather than conventional therapy significantly prevents and reduces the acute and long-term neurological complications for these patients.

#### 4.2.1. Proton Beam Therapy

Proton beam therapy is a modern form of radiotherapy that uses protons. Proton beams have a sharper gradient and allow a precise delivery of radiation, reducing significantly the damage of tumor-surrounding healthy structures. The neurological side effects include neurocognitive deficit, which is reduced compared with traditional radiotherapy [219]. This radiation method is particularly efficient in GB treatment. For instance, in patients with LGG, the use of proton beam therapy reduced neurocognitive complications [220]. The method is also superior to conventional radiotherapy in patients with HGGs, even those with anterior neurocognitive problems [221]. In 2023, Petrucelli et al. compared proton therapy with photon therapy in patients with BTs [222]. He reported a reduced neurocognitive impairment in patients who received proton beam therapy. The study had limitations because it was in silico and produced comparative data only, and the sample size was small [222]. In another study, proton beam therapy was compared with fractionated radiation therapy in patients with GBs. The acute toxicities were similar, but the main limitation of this study was he lack of an established method for distinguishing tumor recurrence from radiation necrosis [223]. There are no differences between the two radiation methods when talking about pseudoprogression in grade II and III gliomas [224] (Figure 2).

Regarding meningiomas, this irradiation method provided tumor control and acceptable toxicity in low-grade or complex meningiomas. The study had limitations because it was single-centered and retrospective, it had imaging limitations, and the meta-analysis demands caution [225]. Unfortunately, the method is limited by high cost.

#### 4.2.2. Stereotactic Radio Surgery (SRS)

Stereotactic radiosurgery (SRS) is a modern method that is less invasive than surgery and enables radiation delivery with a greater therapeutic index compared to conventional external beam radiation therapy (EBRT). It allows for the delivery of a highly conformal dose of ionizing radiation, but with the advantage of minimal radiation exposure to surrounding healthy tissues. It is used both for GB and meningioma treatment, reducing neurological acute and long-term complications when compared with other radiation methods [226,227,228,229]. Regarding GBs, the use of preoperative stereotactic radiosurgery decreases dose delivery to nearby healthy tissues and lowers the risk of treatment-related toxicities like radio necrosis. It also reduces the risk of leptomeningeal disease [230,231] (Figure 2).

#### 4.2.3. Intensity-Modulated Radiation Therapy (IMRT)

Intensity-modulated radiotherapy (IMRT) is a method of radiation characterized by a minimal radiation dose to surrounding normal tissues and critical structures of BTs [232]. This method also allows radiation to penetrate specific anatomical areas simultaneously, delivering radiation at a larger target volume [233] (Figure 2).

#### 4.2.4. Intraoperative Radiotherapy (IORT)

IORT is based on the precise delivery of ionizing radiation to the tumor during the neurosurgery procedure. Nowadays, self-shielding mobile linear accelerators and miniaturized low-kV X-ray IORT devices are used [234]. The radiation doses delivered to the tumor are precise, and include high-dose electrons and low-dose X-rays. Therefore, the residual tumor cells at the resection cavity margins should be destroyed [235]. The advantage of this method is the high doses of radiation administered to the tumor or tumor bed with minimal exposure to radiation of the adjacent tumor tissue. Other advantages of this method are earlier initiation of systemic treatment and shorter treatment time [236]. The improved overall control with IORT reduces the neurological complications after treatment in GB patients [237]. One retrospective study published in 2025 related that patients with HGGs who received IORT during surgery had no severe radiation-related adverse events, such as cerebral radiation necrosis or wound-related complications. No major immediate neurological complications were related. Only a few patients complained of headaches, muscle weakness, seizures, or dysphasia. Delayed neurological complications were present [238]. In a phase 3, open-label, randomized, multicenter trial, INTRAGO-II patients with supratentorial GBs who received IORT presented only a few neurological complications like seizures, but no radiation necrosis or muscle weakness were present [239] (Figure 2).

#### 4.2.5. Strategies to Prevent or Manage Radiotherapy-Induced Brain Edema

The main treatment for brain edema in these patients is osmotherapy with mannitol. Dexamethasone is another option for them, and the first clinical response is usually observed within 24 h. Sometimes, in patients with significant brain edema, the prophylactic use of steroids can be considered [85] (Table 3). Another option for the treatment of post-radiotherapy brain edema in patients with gliomas is *Boswellia serrata*, a dietary supplement derived from the bark of a tropical tree. The boswellic acids have anti-inflammatory properties like inhibition of 5-lipoxygenase, cyclooxygenase-1, nuclear transcription factor kappa B, and tumor necrosis factor alpha. In vitro, *Boswellia serrata* inhibited vascular endothelial growth factor-mediated angiogenesis. One randomized trial proved a 75% reduction of brain edema in patients who received *Boswellia serrata* [240] (Figure 2).

#### 4.2.6. Strategies to Prevent or Manage Early-Delayed Radiation Encephalopathy

Early-delayed radiation encephalopathy usually responds to corticosteroid treatment. Also, antiemetics are useful for nausea and vomiting, and the prognosis is generally favorable. The development of acute encephalopathy is dose-dependent. The modern techniques of radiotherapy make this complication rare nowadays [95] (Figure 2).

#### 4.2.7. Strategies to Prevent or Manage Radiation Necrosis

*Boswellia serrata*, in association with corticosteroids, may be an option for meningioma patients [241].

#### 4.2.8. Strategies to Prevent or Manage the Neurocognitive Impairment

Although current guidelines do not indicate the use of memantine during whole-brain radiation therapy, there is one ongoing phase 3 randomized controlled trial, NCT06275035, the study protocol of which was published in 2026; if the results of this trial are positive, memantine should be considered as a new standard of care for patients who receive craniospinal irradiation and experience altered neurocognitive function [115] (Table 3). Memantine is a low-affinity N-methyl-D-aspartate (NMDA) receptor antagonist. The drug is currently used for dementia patients. There are several mechanisms through which memantine would prevent radiation-induced cognitive decline; among them are the inhibition of inflammation and oxidative stress, and calcium ion influx [242]. Another acetylcholinesterase inhibitor studied in BT patients treated with radiotherapy and with cognitive decline is donepezil, which improved the working memory, motor speed, and dexterity in patients [42]. Methylphenidate was also studied in patients receiving whole-brain radiation [243].

Celecoxib, a cyclooxygenase-2 (COX-2) inhibitor, inhibits apoptosis and reduces the levels of thromboxane B2 as well as 6 keto Prostaglandin F1α (PGF1α) in irradiated human endothelial cells [88]. Therefore, the drug has protective effects on the human brain microvascular endothelial cells against radiation.

Erythropoietin reduces apoptosis and inhibits inflammation. In rats, administration of the drug proved to be protective against memory deficits produced by irradiation [244].

Proton beam therapy also spares normal brain tissue from radiation damage and therefore reduces the neurocognitive impairment [219].

Peroxisome proliferator-activated receptor (PPAR) gamma agonists are ligand-inducible transcription factors. Until now, there were three described isoforms of PPAR: PPAR-α, PPAR-β/δ, and PPAR-γ. PPAR-β/δ is the prevalent isoform in neurons. PPAR-α is expressed at very low levels, predominantly in astrocytes. PPAR-γ expression in the brain is mainly connected with neurodegeneration and inflammation [245]. Pioglitazone, a PPAR agonist, can be protective against radiation therapy in BT patients [246].

Edaravone (EDA) is a free radical scavenger, capable of removing toxic oxygen free radicals that cause brain injury and inhibit oxidative damage of brain cells, vascular endothelial cells, and nerve cells, and improving the role of nerve dysfunction. It has good therapeutic effects on radiation-induced brain injury by diminishing inflammation and oxidative stress [247].

Stem cell therapy, the efficiency of which is up to now proven mainly in preclinical studies, but with encouraging results, seems to be a viable solution to combat radiation-induced neurogenesis damage. Animals exposed to radiation and transplanted stem cell-derived oligodendrocyte progenitors in their corpora callosa, experienced improved cognitive functions like memory and learning capacity, and hippocampal inflammation was also reduced [248,249].

Cognitive training programs have also had a positive impact on some cognitive functions. In BT patients, the use of computer-based cognitive interventions improved cognitive domains like attention and working memory [250] (Figure 2).

### 4.3. Strategies to Prevent or Manage Chemotherapy Complications

#### 4.3.1. Strategies to Prevent or Manage Steroid-Induced Myopathy

The mainstay treatment of steroid-induced myopathy is corticosteroid discontinuation. The monitoring of adrenal insufficiency is also advised. If corticoid discontinuation is not possible, then the replacement of fluorinated glucocorticoids with non-fluorinated glucocorticoids, such as dexamethasone with prednisone or hydrocortisone, should be considered. Phenytoin is thought to facilitate the hepatic metabolism of dexamethasone. Therefore, the association of dexamethasone with phenytoin reduces the risk of steroid-induced myopathy. The non-daily dosing regimen of corticoids is another option. Physical therapy should also be considered for both prevention and treatment [143] (Figure 2).

#### 4.3.2. Strategies to Prevent or Manage CIPN

No preventive or curative treatment is available. Regarding symptomatic treatment, duloxetine can be recommended [251].

#### 4.3.3. Strategies to Prevent or Manage PRES

PRES after bevacizumab administration indicates the retraction of bevacizumab administration in BT patients that develop this complication. If the patient presents seizures, anticonvulsant administration is also recommended [252].

## 5. Future Directions

Even nowadays, BT diagnosis and treatment remain a challenge for specialists. Usually, the diagnosis is late and the treatment options limited, especially in GBs. The significant tumor heterogeneity also contributes to this failure. Glioma classification and prognostics are significantly improved with the use of molecular markers like IDH1/2 mutations, MGMT promoter methylation, TERT alterations, and 1p/19q co-deletion. Beyond these already used biomarkers [253], GBs are characterized by complex genetic alterations known to contribute to tumor initiation and progression, but also resistance to treatment. These alterations usually affect TP53, PTEN, CDKN2A, and EGFR [254]. Therefore, the molecular profiling of surgical or biopsy specimens can improve the diagnosis, prognosis, and treatment options, including toxicity prediction, for these patients. Other tumor-related biomarkers like circulating tumor DNA (ctDNA), cell-free DNA (cfDNA), circulating tumor cells (CTCs), extracellular vesicles (EVs), and microRNAs can be determined from samples of cerebrospinal fluid [254].

AI is a valuable tool nowadays for both neuro-oncologists and radio-oncologists, improving their capabilities to realize integrated diagnosis, a precise prognosis, and assistance in establishing a personalized treatment with lower toxicity. With AI’s help, neuroradiologists can better exploit the MRI images in order to realize tumor segmentation and identification of tumor molecular subtypes. They can also determine the necrotic intratumoral regions or realize quantitative measures [255]. Also, with AI’s help, neurosurgeons can better determine surgical margins and have real-time diagnosis information and guidance which improves surgical precision and diminishes complications [256]. AI’s assistance can also improve the neuropathologists’ diagnoses by providing automatic measures, aiding in tumor classification and grading, and providing better analysis of cellular and tissue structures through histo-molecular classification. AI’s valuable help with biomarker identification or molecular pathway detection provides a diagnosis assistant and a better prediction of the treatment response [257].

Precision neuro-oncology includes treatment choices that are determined by histological classification and biological susceptibility phenotypes like the tumor microenvironment, epigenetic control, metabolic tumor changes, or immune patterns. The following therapies for GBs are studied: IDH-targeted therapy, EGFR-directed therapies, PI3K/AKT/MTOR inhibition, immune checkpoint inhibitors, adoptive cell therapies, oncolytic virotherapies, personalized cancer vaccines, adaptive radiotherapy, and the improved drug delivery through nanotechnologies [258].

## 6. Conclusions

The management of patients diagnosed with BTs is complex and depends on how complications of tumors or tumor treatment are recognized, treated, and even prevented. Even nowadays, despite new therapeutical strategies, the overall survival and quality of life of these patients, especially those with GBs, remain poor. Collaboration between neuro-oncologists and other services like palliative care specialists can improve the discovery of complications of the disease itself, as well as the side effects of various therapies. However, the management of complications like pseudoprogression, refractory epileptic seizures, or cognitive decline faces major hurdles in these patients. Safely controlling epileptic seizures, effectively treating cognitive decline, and reducing steroid administration side effects, along with palliative support, are the most important unmet challenges in the management of neurological complications. Unfortunately, even nowadays there is an overlap between tumor progression and treatment toxicity, and a delicate balance between aggressive disease control and the preservation of patient quality of life.

## Figures and Tables

**Table 1:** Diagnosis and treatment of neurological complications of primary BTs.

Neurological Complication	Diagnosis	Treatment	Level of Evidence
Brain edema	MRI of the head CT scan of the head	Steroids—drug of choice [2] Bevacizumab—did not improve overall survival [18,19] Cediranib—promising results in preclinical studies [20] Celecoxib—although promising in preclinical studies, showed significant adverse effects in clinical trials [22] TGN-020 or AER-271—have the capacity to reduce the cerebral edema in animal models [23]	Guideline recommendation Retrospective study Preclinical study Clinical trial Preclinical study
Seizures	Electroencephalography (EEG)	Surgery, radiotherapy, and chemotherapy Anticonvulsivants Levetiracetam Lamotrigine Valproic acid Enzyme-inducing antiepileptic drugs should be avoided [2]	Guideline recommendation
Stroke	Clinical evaluation MRI of the head—for ischemic stroke CT scan of the head—for hemorrhages	Thrombocytopenia correction with platelet infusion Elevated INR with fresh frozen plasma, vitamin K, prothrombin complex concentrates, activated PCC FEIBA, or recombinant activated factor VIIa. Thrombolysis for ischemic stroke is discussed [39]	Retrospective study
Moya-Moya vasculopathy	Cerebral angiography—especially in the case of unilateral lesions or lesions complicated by atherosclerosis MRI of the head	Bevacizumab should be avoided [41]	Guideline recommendation
Neurocognitive impairment	Clinical assessment Neuropsychological testing Imaging	Donepezil Memantine Methylphenidate and Modafinil [42,43,44] Cognitive rehabilitation	Randomized clinical trial Pilot clinical study

**Table 2:** Neurological complications of primary BT surgery.

Neurological Complication	Incidence	Treatment	Level of Evidence
Sinus venous thrombosis	5–12% [61]	Heparin Oral anticoagulation [62]	Guideline recommendation
Brain ischemia	21% of GBs 43% of meningiomas [63]	Thrombolysis is ill-indicated	Guideline recommendation
Brain hemorrhage	2–23% of meningiomas [71] 7.7% gliomas [69]	Conservative monitoring in Intensive Care Unit Blood pressure management Surgical evacuation	Guideline recommendation
Epiletic seizures	2.9–54.3% intraoperative seizures [76]	Antiepileptic drugs Antiepileptic drugs as primary prophylaxis is not recommended	Guideline recommendation
Hydrocephalus	3.6% of GBs [82] 13.3–16.6% of meningiomas [83]	Ventriculoperitoneal shunt Endoscopic procedures	Guideline recommendation

**Table 3:** Neurological complications after primary BT radiotherapy.

Neurological Complication	Incidence	Treatment	Level of Evidence
Brain edema	5.5% of meningiomas [94]	Mannitol Dexamethasone Prophylaxy with corticosteroids before radiotherapy [123]	Guideline recommendation
Acute radiation encephalopathy	Nowadays almost nonexistent	Surgical removal of tumor Dexamethasone [95]	Guideline recommendation
Early-delayed radiation encephalopathy	3.16% of HGGs [97]	Dexamethasone [124] Bevacizumab [125] Nerve growth factor [126]	Randomized clinical trial Prospective study Randomized clinical trial
Pseudoprogression	GB—up to 39.7% [102] Meningioma—6% [103]	Steroids [98]	Retrospective study
Radionecrosis	GB—21% [110] Meningiomas—4% [113] to 15% [114]	Corticotherapy Surgical decompression	Guideline recommendation
Cognitive decline	50% of patients with BTs [118]	Memantine	Guideline recommendation
Radiation-induced vasculopathy	Ischemic stroke—55% in BT children [127]	Thrombolysis is ill-advised	Guideline recommendation
Radiation-induced cavernomas	23% astrocytoma, 1 glioma [128]	Surgical resection Radiosurgery	Guideline recommendation
SMART syndrome	Case series [129]	No actual treatment	Guideline recommendation

**Table 4:** Neurological complications of primary BT chemotherapy.

Neurological Complication	Drug	Drug Dosage	Drug Mode of Administration	Studies on Primary Brain Tumors
CIPN	Vincristine	2 mg days 8 and 29 in PCV protocol	i.v.	Neurotoxic effect including CIPN [159,160]
CIPN	Larotrectinib	50–100 mg twice daily	oral	Neurotoxic effect including CIPN [168,169]
CIPN	Entrectinib	400–600 mg daily	oral	Neurotoxic effect including CIPN [168,173,174]
Neurocognitive impairment	Temozolomide	75 mg/m^2^ up to 200 mg/m^2^ daily	oral	Cognitive decline [175,176,177]
Neurocognitive impairment	Bevacizumab	5–15 mg/kg every 2 weeks	i.v.	Cognitive decline [178,179]
Neurocognitive impairment	Regorafenib	160 mg/day for 3 weeks	oral	Cognitive decline [180]
Neurocognitive impairment	Nitrosureas	150 mg/m^2^	i.v.	Cognitive decline [181]
Posterior reversible encephalopathy syndrome (PRES)	Bevacizumab	5–15 mg/kg every 2 weeks	i.v.	PRES [182]

## Data Availability

No new data were created or analyzed in this study. Data sharing is not applicable to this article.
